# Enhancing Africa's stroke workforce to address the stroke burden: a proposal from the African's stroke organization's educational and training committee

**DOI:** 10.3389/fstro.2025.1611194

**Published:** 2025-06-18

**Authors:** Paul Bolaji, Ebenezer Ad Adams, Rufus Akinyemi, Mayowa Owolabi, Foad Abd-Allah

**Affiliations:** ^1^Dorset County Hospital, Dorchester, United Kingdom; ^2^African Stroke Organisation, Ibadan, Nigeria; ^3^Institute for Advanced Medical Research and Training, College of Medicine, University of Ibadan, Ibadan, Nigeria; ^4^Department of Neurology, Kasralainy School of Medicine, Cairo University, Cairo, Egypt

**Keywords:** stroke, education, Africa, capacity building, training

## Abstract

Stroke is the second leading cause of death and disability in Africa, disproportionately affecting individuals in their most productive years and placing an immense socio-economic burden on families and healthcare systems. Despite the growing stroke burden, Africa faces a severe shortage of trained stroke specialists, with only three neurologists per 10 million people compared to up to 900 per 10 million in high-income countries. This gap has led to inadequate acute management, limited rehabilitation services, and poor long-term outcomes. To address this crisis, the African Stroke Organization (ASO) established an Education and Training Committee focused on developing a structured stroke education framework. This proposal outlines ASO's strategic initiatives to build stroke care capacity through research, professional training, service development, and advocacy. By implementing a comprehensive stroke education program-including online courses, hands-on workshops, conference-based training, and a future summer school, ASO aims to train 5,000 by 2030. Through this initiative, ASO seeks to empower African healthcare professionals, reduce disparities in stroke care, and ultimately improve stroke outcomes across the continent. This proposal presents the rationale, objectives, and implementation strategies for the ASO Stroke Education Program and calls for international collaboration to support this critical effort.

## Introduction

Africa is a home to more than one billion population facing an escalating cardiovascular disease including stroke crisis (Moran et al., 2013; Owolabi, 2011). Stroke is the second leading cause of adult mortality in the region with incidence rates range from 150 to 316 per 100,000 population annually-particularly in Sub-Saharan Africa, significantly higher than many high-income countries with an average estimate up to three million strokes in Africa (Adoukonou et al., 2021; Akinyemi et al., 2021).

Stroke disproportionately affects individuals in their most productive years (ages 30–60), creating an enormous socio-economic burden on families and the healthcare system (Thayabaranathan et al., 2022) A large majority of stroke survivors in Africa face long-term disability due to delayed diagnosis, inadequate acute management, and limited rehabilitation services (Kayola et al., 2023; WHO, 2024).

Despite the high disease burden, stroke care in Africa remains critically underdeveloped (Sarfo et al., 2023; Walker, 2022). Only 10% of stroke patients receive guideline-based acute interventions such as thrombolysis or thrombectomy due to resource constraints and a lack of trained personnel (Owolabi et al., 2015). Access to essential diagnostic imaging, such as CT and MRI, remains scarce, with some countries having fewer than one CT scanner per million people, compared to over 10 per million in high-income regions (Hricak et al., 2021).

There are also challenges with stroke rehabilitation in Africa. Stroke Rehabilitation seeks to optimize functioning of people with impairments and includes a range of specific health services—diagnosis, treatment, surgery, assistive devices, and therapy. Evidence on access to rehabilitation services for people with disabilities in low- and middle-income countries (LMICs) is limited (Bright et al., 2018).

Stroke rehabilitation is an integral part of universal health coverage, and access to it is a human right. Poor access to rehabilitation services is associated with less attention given to rehabilitation by governments, which leads to less funding, negative cultural and social beliefs, fewer rehabilitation centers, poorly equipped rehabilitation units, failure of health systems, lack of training to rehabilitation practitioners, and logistical and financial constraints (Cyuzuzo et al., 2025).

A key contributor to this disparity is the severe shortage of stroke specialists. Africa has only three neurologists per 10 million people, compared to up to 900 neurologists per 10 million in Europe-a 300-fold difference (Steinmetz et al., 2024). This shortage perpetuates a cycle where inadequate training opportunities lead to professional migration (“brain drain”), further weakening stroke care capacity (Roushdy et al., 2022).

Recognizing these challenges, three leading African Neurologists and Professors; Akinyemi, Abd-Allah, and Owolabi and a Rehabilitation expert-Ebenezer Ad Adams with other eminent African Neurologists established the African Stroke Organization (ASO) in 2020. ASO aims to bridge this gap by fostering stroke education, research, service development, and advocacy (Akinyemi et al., 2020).

## The African Stroke Organization framework

The African Stroke Organization (ASO) framework is built on four key pillars (Akinyemi et al., 2020):

1. **Research:** advancing stroke research to generate African-specific data for policy development and clinical practice.
2. **Capacity building**: training African stroke professionals to improve stroke prevention, acute management, and rehabilitation.
3. **Development of stroke services** (**stroke quadrangle):** strengthening infrastructure and care models to enhance service delivery (Olatunji et al., 2023).
4. **Advocacy, awareness, and collaboration**: engaging stakeholders to prioritize stroke care and influence policy changes.

More recently, in 2022, the first African Stroke Leaders' Summit (ASLS) was held through the Africa–UK Stroke Partnership (AUKSP) Project. During this event, 52 stroke leaders from across all regions of Africa participated in a 2-day plenary session aimed at developing strategies to improve stroke care. The discussions focused on four key domains: stroke services, stroke training and capacity building, research, and stroke advocacy. Stroke Education and Capacity Building was highlighted as a priority to improve stroke care in Africa (Akinyemi et al., 2025).

To achieve the Capacity Building goal of the African Stroke Organization (ASO), Five Faculty members including Three Professors of Neurology-Foad Abd-Allah, Rufus Akinyemi, and Mayowa Owolabi; a Consultant Stroke Physician-Paul Bolaji, and a Rehabilitation expert-Ebenezer Ad Adams came together to develop a blueprint for the African Stroke Organization Education Framework.

They recognized the urgent need to enhance stroke care capacity in the region. By providing healthcare providers with the necessary tools, knowledge, and resources, the ASO aims to improve stroke outcomes significantly.

This proposal details ASO's Education Framework, designed to train a new generation of African stroke care professionals and improve stroke outcomes across the continent (Omaswa et al., 2018).

## Capacity building priorities of ASO Education Framework

The ASO Education Framework prioritizes stroke training in three key areas:

- Training stroke professionals: establishing structured educational programs for physicians, nurses, and allied healthcare workers.
- Addressing stroke disparities: developing curricula tailored to the specific needs of African populations and individuals of African ancestry.
- Research training: enhancing capacity for basic, clinical, translational, behavioral, and population-based stroke research.

## Vision of the African Stroke Organization (ASO) Stroke Education Program

1. **Empowering African stroke care providers:** equip the next generation of neurologists, stroke physicians and other stroke health professionals of the Stroke Multidisciplinary team with evidence-based and actionable knowledge tailored to Africa's unique stroke burden.
2. **Driving innovation**: inspire early-career professionals to develop Afrocentric, innovative strategies to address stroke care gaps (Olatunji et al., 2023).
3. **Fostering collaboration**: unite stroke care providers across all regions across African countries; both anglophone and francophone countries to tackle shared challenges and foster regional collaborations along with government authorities to shape polices in stroke care, education, prevention and research.

## Aim and objectives of the African Stroke Organization (ASO) Education Program

1. To equip 1,000 African Stroke Care Providers with up to date, relevant, and insightful online stroke education program by the year 2026.
2. By 2030, to have delivered majority of the multi-tiered face to face and online quality education program to 5,000 African Stroke Care Providers.
3. By 2035: To have started ASO Stroke Fellowship Program and reached more than 10,000 African Stroke Care providers through the multi-tiered face to face and online education program.

## ASO Stroke Education Program: implementation plan

ASO proposes a multi-tiered educational approach to achieve its training goals.

1. **Online continuing medical education and professional development**
  - Under the monthly continuing professional development... ensure certification for stroke health professionals like doctors, occupational therapists, physiotherapists, speech and language therapists, stroke nurses, etc.
  - Monthly webinars providing accessible, cost-effective stroke training.
2. **In person stroke workshop**
  - Regional 2-day hybrid workshops focused on hands-on stroke management training.
  - Targeted at healthcare providers within specific localities to reduce travel costs.
3. **Pre-conference courses at ASO conferences**
  - Intensive training courses held in conjunction with ASO's annual conference.
  - Designed to engage a broad audience of stroke care providers and researchers.
4. **ASO summer school (future initiative)**
  - A specialized training program for early-career stroke professionals.
  - To be implemented once the regular ASO Education Program is well established.
  - ASO Summer School will involve an intense certification course duration 2–4 weeks on Stroke Mgt.
5. **ASO stroke fellowship program (future initiative)**
  - Stroke Fellowship program will be targeted at trainees or consultant in Neurology or Internal Medicine who want to sub-specialize in stroke medicine.
  - This Fellowship program will be a 12–24 months intense program, designed to train the next generation of African Stroke specialist in cutting edge and evidence-based medicine, and skills in all aspects of stroke care from Prehospital care, primary and secondary prevention, Hyperacute stroke care, Acute stroke care and stroke rehabilitation.

## Projected impact of ASO Stroke Education Program

### Strengthening stroke care capacity

Regular training will equip healthcare professionals with evidence-based stroke prevention, diagnosis, treatment, and rehabilitation strategies, improving patient outcomes.

### Encouraging interdisciplinary collaboration

By fostering collaboration among neurologists, nurses, therapists, and other professionals, ASO's training programs will promote a more integrated approach to stroke care.

### Reducing brain drain and promoting local retention

By providing region-specific education, ASO aims to empower local professionals, reducing the need for migration in search of training opportunities.

### Supporting career development

ASO's structured educational programs will enhance professional development through certifications, mentorship, and networking opportunities, strengthening the African stroke workforce.

## Conclusion

The African Stroke Organization's (ASO) Education and Training Committee is committed to addressing the critical shortage of trained stroke professionals in Africa. Through a structured and scalable education framework, ASO aims to bridge the knowledge gap, enhance stroke care capacity, and ultimately reduce the burden of stroke-related disability and mortality on the continent (Akinyemi et al., 2021).

By equipping 1,000 stroke care providers by 2026 and expanding to 5,000 by 2030, ASO's initiatives-including online education, hands-on workshops, conference courses, and future summer school-will foster interdisciplinary collaboration, encourage local retention, and drive innovation in stroke care tailored to Africa's unique challenges.

Investing in stroke education is not just about training individuals-it is about transforming healthcare systems, empowering communities, and ensuring that high-quality stroke care is accessible across Africa. Through strategic partnerships, ongoing research, and sustained advocacy, the ASO Education Framework will play a pivotal role in shaping the future of neurology and stroke care on the continent.

## Data Availability

The original contributions presented in the study are included in the article/supplementary material, further inquiries can be directed to the corresponding author.
